# Investigating the contribution of tongue strength measurement in determining pre‐clinical Alzheimer's disease pathology in older adults

**DOI:** 10.1002/alz70856_105356

**Published:** 2026-01-08

**Authors:** Getachew Yideg Yitbarek, Jane E. Alty, Eddy Roccati, Katherine Lawler, Lynette R. Goldberg

**Affiliations:** ^1^ Wicking Dementia Research and Education Centre, University of Tasmania, Hobart, TAS, Australia; ^2^ School of Medicine, University of Tasmania, Hobart, TAS, Australia; ^3^ Royal Hobart Hospital, Hobart, TAS, Australia; ^4^ La Trobe University, Melbourne, VIC, Australia

## Abstract

**Background:**

Alzheimer's disease (AD) accounts for 60‐70% of dementia cases. Pathological changes begin 15‐20 years before symptoms appear. Identifying non‐invasive biomarkers to detect these changes are required. Gait speed, hand movements and grip strength have been associated with cognitive decline. A recent review also documented associations between cognitive decline and decreased tongue strength. The current study investigated the association of tongue strength with grip and pinch strength and a blood biomarker of AD (plasma *p*‐tau 181) in cognitively healthy older (adults ≥ 50 years).

**Method:**

This cross‐sectional study involved participants from the ISLAND Project in Tasmania who completed motor assessments and blood samples analysed for *p*‐tau 181. Pearson's correlation coefficients determined the correlation of tongue strength with pinch and grip strength across different *p*‐tau 181 tertiles. One‐way ANOVA and Tukey's HSD tests were used to compare and identify groups with significant mean Skeletal Muscle Mass Index (SMI) differences across tongue tertiles. A multivariable linear regression model was fitted, adjusted for possible confounders, determined after Directed Acyclic Graph analysis.

**Result:**

158 participants (75 % female) with a mean age of 69.32 (±6.7) years were recruited. Mean tongue strength was 48.09 kPa (±10.49) and mean *p*‐tau 181 was 1.37 pg/ml (±0.57). Participants with normal tongue strength had lower mean *p*‐tau 181 levels (1.36 pg/ml) than those with lower tongue strength (1.43 pg/ml). The mean SMI of participants in the highest tongue tertile (7.8 kg/m^2^) was higher than those in the second tertile (7.03 kg/m^2^) (*p* = 0.0329, 95% CI (0.06,1.47)). Tongue strength was positively correlated with pinch (*r* = 0.45, *p* <0.001) and grip strength (*r* = 0.35, *p* = 0.01), established markers of cognitive decline. The correlation was more pronounced as *p*‐tau 181 levels increased. No mean tongue strength difference or associations were observed across the *p*‐tau 181 tertiles or in the multivariable regression analysis.

**Conclusion:**

The higher mean *p*‐tau 181 level observed in individuals with lower tongue strength, along with the effect of *p*‐tau 181 level on tongue strength with grip and pinch strength correlations, speaks to the contribution of tongue strength measurement in determining preclinical AD pathology. Future longitudinal studies are needed to better understand the observed associations.

